# Facial emotion recognition in children with attention deficit hyperactivity disorder

**DOI:** 10.1080/08039488.2024.2403589

**Published:** 2024-09-18

**Authors:** Markus Varheenmaa, Soili M. Lehto, Patrizia Rizzo, Hans-Christoph Steinhausen, Renate Drechsler, Anna-Katharine Brem

**Affiliations:** a Department of Psychiatry, Institute of Clinical Medicine, University of Eastern Finland, Kuopio, Finland; b Institute of Clinical Medicine, University of Oslo, Oslo, Norway; c R&D department, Division of Mental Health Services, Akershus University Hospital, Lørenskog, Norway; d Department of Psychiatry, University of Helsinki, Helsinki, Finland; e Clinic for Child and Adolescent Psychiatry and Psychotherapy, Psychiatric University Hospital, Zurich University, Switzerland; f Clinical Psychology and Epidemiology, Department of Psychology, University of Basel, Switzerland; g Department of Child and Adolescent Psychiatry, University of Southern Denmark, Odense, Denmark; h Centre for Healthy Brain Ageing, Department of Psychological Medicine, Institute of Psychiatry, Psychology and Neuroscience, King’s College London, London, UK; i University Hospital of Old Age Psychiatry, University of Bern, Bern, Switzerland

**Keywords:** ADHD, facial affect recognition, emotion processing

## Abstract

**Objective:**

Attention Deficit Hyperactivity Disorder (ADHD) is defined as a persistent pattern of inattention and hyperactivity-impulsivity that interferes with functioning anofd development. Increased emotional reactivity and impaired emotion regulation are established findings in children with ADHD. Impairments in executive functions such as impulse control and working memory, in turn, have also been suggested to have a negative effect on emotion recognition. However, studies exploring suspected deficits in the ability to recognise facial emotions in ADHD have to date yielded controversial results. We sought to clarify the mechanism of possible emotion recognition dysfunction in children with ADHD.

**Methods:**

Sixty-one children diagnosed with ADHD (aged 10.36 ± 1.89 years) and a control group (*N* = 78; aged 9.6 ± 1.8 years) were evaluated with questionnaires and computerized tests for cognitive and facial emotion recognition capacity.

**Results:**

The ADHD group displayed more behavioural issues and performed worse in cognitive tests compared to the control group. Group status (i.e. ADHD vs. control group) did not predict facial emotion recognition when controlled for age, IQ and sex in linear regression models. Performance in Divided Attention predicted facial emotion recognition in linear regression in the ADHD group.

**Conclusions:**

Individuals with ADHD showed facial emotion recognition capacity similar to a typically developing control group. Good performance in a cognitive test assessing divided attention predicted capacity for facial emotion recognition, but only in the ADHD group.

## Introduction

Attention-Deficit Hyperactivity Disorder (ADHD) is a common childhood psychiatric disorder with a prevalence rate of 5%-7% worldwide [1]. Its core symptoms are impulsivity, inattention, and hyperactivity [2]. In children with ADHD, increased emotional reactivity and impaired emotion regulation are established findings [3]. However, to date, studies exploring suspected deficits in their ability to recognise facial emotional expressions have yielded controversial results [4,5]. Current research in this area has three main directions.

The first line of research highlights deficits in recognising facial expressions as a secondary negative effect. This effect results from primary cognitive impairments and cognitive function regulation impairments varyingly observed in ADHD populations, or the core symptoms themselves. For example, Cadeskyet al., (6) suggested already 20 years ago that deficits in the encoding of stimuli affected by inattention, and a general impairment in regulatory processes result in adversity in interpreting emotion [6]. Further research on cognitive processes influencing core ADHD symptoms and facial emotion recognition has been suggested by studies that observed facial emotion recognition deficit in ADHD [7–10].

The second line of research argues that emotion recognition impairments linked with ADHD are independent of cognitive functions. Sjöwall et al., 2013 [11] reported children with ADHD to show inferior performance to typically developing peers in recognizing five basic emotions—happy, sad, anger, fear, and surprise. They found that facial anger recognition, along with regulation of anger and happiness/exuberance, discriminate groups farther than cognitive functions such as working memory, inhibition, or shifting do. Previously it was found that 85% of the variance between the ADHD and the control groups was explained by a facial affect recognition test [12]. Furthermore, boys with ADHD faced challenges linking appropriate emotions to pictures depicting social situations, which were simultaneously described in short sentences. These observations remained unaltered when impulsivity was considered [13]. A study utilizing a morphing task found children with ADHD to exhibit lower accuracy rates across all emotional expressions without any evidence for impaired perceptual sensitivity [14]. Two recent studies furthermore found impaired facial emotion recognition processing in ADHD [15,16].

Finally, in studies constituting the third line of research, facial emotion recognition capacity in ADHD and control children has been found to be equal [17–23]. For example, no difference in facial emotion recognition performance emerged between ADHD and typically developing children, while compared together with autistic children [18]. Additionally, impaired facial emotion recognition was present in ADHD with comorbid oppositional defiant disorder (ODD) or opposition in general. In the absence of these, however, ADHD groups displayed no difference to controls [20]. Similarly, Schwenck et al., [22], utilizing a morphing task, observed no difference in facial emotion recognition performance between ADHD children and a control group, when ODD and conduct problems were controlled for [22]. Nonetheless, in a recent study, facial emotion recognition was found intact in children with ODD or disruptive mood dysregulation disorder (DMDD) while being positively correlated with cognitive flexibility. A majority of the children with DMDD also had ADHD [24]. Additionally, in a series of studies, Wells and colleagues have found working memory to be related to emotion recognition in general and ADHD children performing equally to controls in emotion recognition tasks [23,25].

Adjusting behaviour to fit a myriad of social situations demands recognizing affective states conveyed by the facial expressions of others [26]. Recognition involves brain areas relevant for visual processing (i.e. occipital and temporal regions), face processing (i.e. fusiform face area), and important areas relevant for the processing of facial emotional expression –the orbitofrontal cortex and the amygdala. All these areas together modulate face perception and trigger associated knowledge *via* projections to other areas of the neocortex and hippocampal areas, inducing emotional responses. Thus, the brain generates knowledge about the emotional states of others by simulating them [27,28].

Several attention and executive functions, such as working memory [29,30], vigilance, divided attention, inhibitory control, incompatibility, flexibility, as well as the integration of sensory information have been reported to be impaired in ADHD [31]. Sustained attention, inhibitory control [9], and distractibility [18] have furthermore been associated with impairments in emotion recognition in ADHD. Nevertheless, further research is needed, as another study conducted in children with ADHD reported no association between emotion recognition deficits and sustained attention or distractibility [10]. Cognitive tests of attention and executive control discriminate children with ADHD from typically developing children on the group level, but individual cognitive profiles are extremely heterogeneous [32,33,34]

We investigated facial emotion recognition in children with ADHD and a control group and sought to clarify the mechanisms of possible emotion recognition dysfunction in ADHD children, and alterations in underlying behaviour. We used facial emotion recognition tasks specifically tailored for children and measured cognition (alertness (tonic), divided attention, inhibition, incompatibility, flexibility, distractibility, and sustained attention) through computerized cognitive tests in both study groups. Inattention, impulsivity, and hyperactivity were assessed through questionnaires.

We hypothesized that typically developing controls outperform children with ADHD in facial emotion recognition, and core ADHD symptoms or deficits in cognitive performance offer no explanation for this phenomenon.

## Methods

### Participants

Sixty-one children with ADHD, aged 10.36 (± 1.89) years and representative of the local population, were recruited *via* the Department for Child and Adolescent Psychiatry, University of Zurich, Switzerland, which included eight (in or outpatient) clinics in the Canton of Zurich, in urban as well as rural areas. Prior to entering the study, all but three participants were clinically diagnosed with ADHD in child and adolescent psychiatry by mental health specialists according to ICD-10. All ADHD diagnoses were confirmed according to the Diagnostic and Statistical Manual of Mental Disorders, 4th Edition (DSM-4) upon entering the study. Children with comorbid psychopathological diagnoses (autism, anxiety, depression, OCD) or brain lesions, inability to recognize faces, and an intelligence quotient (IQ) < 85 were excluded. Individuals with concurrent minor ODD/CD or concurrent diagnoses of learning disorders (e.g. dyslexia) were allowed in the study sample. Children with ADHD taking stimulant medication had to discontinue medication at least 48 h before testing. The control group (*N* = 78, age 9.6 ± 1.8 years) was recruited through schools in the greater Zurich and Lucerne area, Switzerland (Table 1). The local ethics committee approved the study protocol and all children and parents gave written informed consent before study onset.

**Table 1.** Demographic and behavioural characteristics of the participants.

	ADHD Mean (SD)	Control Mean (SD)	Test statistics	*p*
Background variables				
	*n* = 61	*n* = 78		
Age	10.4 (1.9)	9.6 (1.8)	−2.45^a^	*0.016*
Female (*n* (%))	10 (16.4)	19 (24.4)	1.32^b^	0.251
IQ	109 (13.4)	115 (15.7)	2.71^a^	*0.008*
Swanson, Nolan and Pelham Questionnaire subscale scores (SNAP)				
	*n* = 56	*n* = 69		
Inattention	16.23 (5.2)	5.10 (4.1)	−13.35^a^	*<0.001*
Hyperactivity/impulsivity	10.90 (6.35) ^c^	3.07 (2.53)	−8.60^a^	*<0.001*
Oppositional defiant	8.88 (5.53) ^d^	4.38 (3.36)	−5.15^a^	*<0.001*
Child behavior checklist T scores (CBCL)				
	*n* = 58	*n* = 70		
Withdrawn	58.69 (8.50)	52.86 (4.30)	−4.75^a^	*<0.001*
Somatic complaints	57.84 (8.50)	53.39 (5.17)	−3.50^a^	*0.001*
Anxious/ depressed	60.72 (10.73)	52.54 (4.46)	−5.43^a^	*<0.001*
Social problems	61.83 (9.96)	53.00 (5.05)	−6.13^a^	*<0.001*
Thought problems	56.93 (8.78)	51.34 (3.30)	−4.60^a^	*<0.001*
Attention problems	65.79 (9.37)	52.81 (4.79)	−9.60^a^	*<0.001*
Delinquent behaviour	61.52 (9.86)	53.06 (5.84)	−5.75^a^	*<0.001*
Aggressive behaviour	62.84 (11.14)	53.70 (6.28)	−5.56^a^	*<0.001*
Internalizing problems	59.31 (11.16)	48.10 (8.12)	−6.38^a^	*<0.001*
Externalizing problems	62.62 (11.63)	48.83 (10.29)	−7.12^a^	*<0.001*
Total score	64.29 (11.79)	48.07 (9.25)	−8.72^a^	*<0.001*
Strengths and difficulties questionnaire subscale scores (SDQ)				
	*n* = 58	*n* = 70		
Emotional symptoms	3.66 (2.78)	1.30 (1.16)	−6.05^a^	*<0.001*
Conduct problems	3.38 (2.36)	1.53 (1.47)	−5.27^a^	*<0.001*
Hyperactivity/inattention	6.95 (1.93)	2.36 (1.99)	−13.15^a^	*<0.001*
Peer relationship problems	3.19 (2.83)	1.11 (1.50)	−5.02^a^	*<0.001*
Prosocial behaviour	6.88 (2.11)	7.66 (2.14)	2.06^a^	*0.041*
Total problems	17.16 (7.64)	6.29 (4.04)	−9.76^a^	*<0.001*
Behavioral rating inventory of executive functions subscale scores (BRIEF)				
	*n* = 58	*n* = 69		
Inhibit	19.33 (5.30)	12.93 (3.15)	−8.07^a^	*<0.001*
Shift	14.41 (3.93)	10.80 (2.76)	−5.89^a^	*<0.001*
Emotional control	20.38 (5.39)	14.59 (4.42)	−6.64^a^	*<0.001*
Behavior regulation index (BRI)	54.12 (12.40)	38.32 (8.95)	−8.09^a^	*<0.001*
Initiate	15.84 (3.43)	11.04 (2.63)	−8.93^a^	*<0.001*
Working Memory	23.07 (4.04)	13.51 (3.70)	−13.90^a^	*<0.001*
Plan/organize	25.98 (5.72)	16.20 (4.63)	−10.47^a^	*<0.001*
Organization of material	13.38 (3.57)	10.67 (3.36)	−4.39^a^	*<0.001*
Monitor	18.24 (3.49)	12.68 (3.39)	−9.08^a^	*<0.001*
Metacognition index (MI)	96.52 (16.63)	64.10 (14.47)	−11.75^a^	*<0.001*
Global executive composite (BRI + MI)	151.54 (25.40)	102.42 (20.84)	−11.93^a^	*<0.001*

^a^Student T-test,

^b^Chi-Square test,

^c^n(ADHD)=55

^d^n(ADHD)=51; SD: standard deviation statistically significant figures are presented in *italics*.

### Study setting

Children with ADHD were tested at the Centre for Child and Adolescent Psychiatry, University of Zurich, Switzerland, the control subjects attended experimental sessions in quiet rooms at their schools. Complete test duration was 3–4 h per child and was divided into 2–3 test sessions. Questionnaires were filled in prior to testing at home or at the test site. Computerized tests of cognition and facial emotion recognition were performed on a laptop computer (screen size 15’).

### Questionnaires

The revision of the ‘Swanson, Nolan and Pelham Questionnaire’ (SNAP) [35] was used to measure ADHD and ODD symptoms and confirm diagnosis. Control children reaching clinical scores on the SNAP (subscale score cutoffs: inattentive type 1.78; hyperactive/impulsive type 1.44; combined type 1.67) were excluded. ‘The Child Behaviour Checklist’ (CBCL) [36] was used to identify problematic behavior that parents have observed in their child. Parents’ report on their child’s executive functioning was acquired *via* the ‘Behavioural Rating Inventory of Executive Functions’ (BRIEF) [37,38]. Parents furthermore were asked to evaluate their child’s psychological adjustment *via* the ‘Strengths and Difficulties Questionnaire’ (SDQ) [39].

### Cognitive tests

IQ was assessed with a short version of the German Wechsler Intelligence Scale for Children (Hamburg-Wechsler-Intelligenztest für Kinder (HAWIK-III) [40], the ability to recognize faces was evaluated with a modified version of the ‘NEPSY – a developmental neuropsychological assessment’ subtest ‘Memory for Faces’ [41]. The ‘Test of Attentional Performance’ (TAP) [42] and the ‘Test of Attentional Performance for Children’ (KITAP) [43] were performed by children above and below 10 years of age, respectively. Both test batteries assess the same cognitive functions; however, the KITAP features a child-friendly design.

The following tasks were administered: Alertness (tonic) where the subject should respond, as quickly as possible by pressing a key, to a cross that appears on the monitor at randomly varying intervals. Divided Attention in which auditory and visual processing must be done simultaneously. Inhibitory Control where predictably occurring stimuli require to react or not to react and attention is directed at them. Incompatibility which tests interference tendency. Flexibility which is a set shifting task. Distractibility which is a continuing decision task presented in the centre of the monitor and where in half of the trials a distractor stimulus appears in the visual field periphery. Sustained Attention which is a task that requires maintaining attention selectiveness over a long period of time. Comparison of a stimulus with a subsequent stimulus is required to determine whether these two stimuli have a predetermined stimulus feature in common.

### Facial emotion recognition assessment

**Figure 1.**
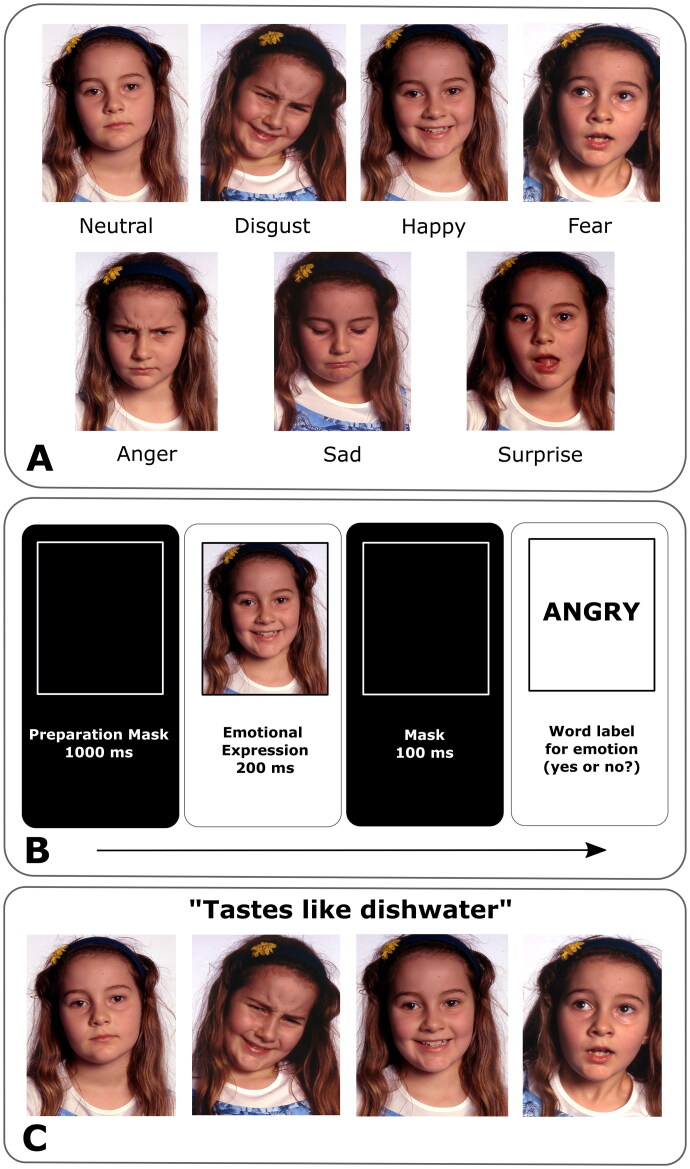
Emotional expression photos and tasks. **(A)** Example set of six facial emotion expressions (disgusted, happy, fearful, angry, sad, surprised) and a neutral expression. Photos were used in the facial emotion recognition task **(B)** and the sentence matching task **(C)** as schematized.

A test battery suitable for assessing emotion recognition in children was developed in collaboration with the Zurich University of the Arts and with child actors from a child theatre group. The test battery comprised photographs of children expressing seven different emotional expressions (Happy, Sad, Angry, Surprised, Fearful, Disgusted and Neutral) (Figure 1). Photographs were first rated by 24 adults in two tests: 1) each photograph had to be assigned to one of seven facial emotion expressions; 2) a set of seven emotions had to be assigned to one of seven emotions. To be included in the final study test set, a minimum of 70% corrects in the first task and 90% corrects in the second task were required. To be included as a distractor item the limits were lowered to 60% and 88%, respectively. In a second step, we designed two computerized emotion recognition tasks (the Facial emotion recognition task and the Sentence matching task).

### Facial emotion recognition task

Facial emotion recognition task comprised a photograph of an emotional expression presented for 200 ms followed by a 100 ms mask followed by an emotion word (auditory presentation and in writing). Each trial was preceded by a 1000 ms preparation mask. Subjects had to indicate by button press whether the word was associated with the depicted facial expression or not (left button press for ‘yes’ or right button press for ‘no’) (Figure 1). Children were familiarized with the material in a practice trial with feedback. No feedback was provided in the main test. The task consisted of 120 trials featuring facial emotion expressions of Happy, Sad, Anger, Surprise, Fear, Disgust and Neutral. Each emotion served as a target twenty times, whereas the expression depicted on the photograph corresponded with the emotion word in half of the trials (e.g. Happy–Happy) and was presented with the other 6 emotions in the remaining trials.

### Sentence matching task

In the Sentence matching task, subjects were to choose one of four facial emotion expressions that best fit a sentence with emotional content (e.g.: ‘The little dog has died.’; ‘I hate cabbage soup.’) (Figure 1). Sentences were presented auditorily and in writing concurrently with the four photographs. Responses were self-paced and registered *via* an external response box with four numbered buttons. The task consisted of 42 trials with facial emotion expressions of Happy, Sad, Anger, Surprise, Fear, Disgust and Neutral. Each emotion served as a target seven times. Children were familiarized with the material in a practice trial with feedback. No feedback was provided in the main test.

### Statistics

Chi-square-tests and Student T-tests were used to examine group differences. All non-normal continuous variables were normalised using natural logarithm for positively skewed or kurtosed variables and negatively skewed or kurtosed variables were squared. Cognitive test results were analysed using the T-values which enabled comparison of younger and older children.

Pearson correlations of questionnaire sum scores, cognitive test results and facial emotion recognition test results were calculated within both groups (i.e. children with ADHD and the control group); furthermore, we examined whether these correlations differed statistically between the two studied groups. Possible group differences between the ADHD group and the control group in combined positive (i.e. Happy, Neutral, Surprised) versus negative emotions (i.e. Sad, Angry, Fearful, Disgusted) recognition capacity were also assessed with the Student T-test.

We calculated the observed effect sizes (Cohen’s d) of group comparison for correct answers and reaction times in the Facial emotion recognition task and the Sentence matching task using G*Power [44].

Linear regression models were used to examine the contribution of group affiliation to performance in cognitive and facial emotion recognition tests. We constructed two separate models for regression analyses. Model 1, the basic model, was adjusted for age, and Model 2 was adjusted for age, IQ and sex.

At the next stage, we ran an additional linear regression model on cognitive tests with observed group differences in the T-test to examine the presence of possible interaction between cognition and group status as predictor for facial emotion recognition capacity (Model 3, adjusted for age, IQ, sex, group and cognitive test x group). Each cognitive test was separately inserted to the model. The impact of cognitive tests displaying a significant interaction term were then further investigated by dividing the dataset based on group status and examining how the performance in cognitive tests displaying a significant interaction with group status predicted facial recognition capacity within ADHD and control groups. These models were adjusted for age, IQ and sex.

## Results

Sex distribution was similar between the two groups, while the ADHD group was slightly older (*p=*.016) and had a lower IQ (*p=*.008) than the control group, which was considered in Models 2 and 3 of the regression analyses. Groups differed significantly in their responses to questionnaires addressing behavioural problems. As expected, the sum scores of the metacognition and behaviour regulation index in the BRIEF, the total problem score in the SDQ, and the T-score total score in the CBCL were significantly increased in the ADHD group (*p_s_*<.001) (Table 1).

### Correlation of emotion recognition with psychopathological, behavioural and cognitive measures

The SNAP Oppositional Defiant Subscale did not correlate with number of correct responses in the Emotion recognition (ADHD r=-0.137, *p* = 0.349; Control r=-0.060, *p* = 0.645) or Sentence matching (ADHD r= −0.139, *p* = 0.335; Control r= −0.034, *p* = 0.797) tasks (Supplementary Table 1). Similarly, the CBCL scales Social problems and Anxious/Depressed was not correlated with the number of correct responses of Emotion recognition (ADHD: Social problems *r*=.129, *p*=.344, Anxious/Depressed *r*=.046, *p*=.736; Control: Social problems r= −0.096, *p*=.460; Anxious/Depressed *r*=.095, *p*=.460). Correlations of emotion recognition with cognitive test results are reported in the Supplementary Materials and Supplementary Table 1.

### Cognitive functions

We observed no differences in reaction time T-values between groups in any of the cognitive tests. However, the ADHD group displayed more commission errors in the majority of cognitive tests (Divided Attention *p=*.002; Flexibility *p=*.003; Incompatibility *p=*.001; Distractibility *p=*.010 and Sustained Attention *p=*.001). Furthermore, in the ADHD group, omission errors were more frequent in the Inhibitory Control (*p<*.001)-test and SD’s were larger in Flexibility (*p=*.003) and Incompatibility (*p=*.001) -tests (Table 2).

**Table 2.** Cognitive performance of study participants.

Variable	ADHD (T-value)	Control (T-value)	Student T-test	*p*
Alertness				
	*n* = 58	*n* = 71		
Median RT	47.68	49.76	1.131	0.261
SD	45.38	47.74	1.142	0.256
Divided attention				
	*n* = 57/54	*n* = 70/71		
Median RT	49.72	49.29	−0.281	0.779
Comission errors	46.85	52.76	3.167	*0.002*
Inhibitory control				
	*n* = 58/59/58/57	*n* = 71/74/71/71		
Median RT	48.24	48.89	0.284	0.777
SD	38.34	41.04	1.616	0.109
Comission errors	46.36	49.47	1.346	0.181
Omissions	42.34	48.53	4.601	*<0.001*
Flexibility				
	*n* = 55/53/55	*n* = 68/65/69		
Median RT	44.84	46.61	1.096	0.275
SD	40.85	46.40	3.777	*<0.001*
Comission errors	46.92	53.33	3.003	*0.003*
Incompatibility				
	*n* = 53	*n* = 57		
Median RT	43.37	44.35	0.478	0.634
SD	39.45	44.42	2.609	*0.010*
Comission errors	46.64	53.92	3.317	*0.001*
Distractibility				
	*n* = 32	*n* = 55		
Median RT	46.23	45.64	−0.260	0.795
Comission errors	49.33	56.32	2.628	*0.010*
Omissions	50.85	51.79	0.513	0.609
Sustained attention				
	*n* = 29	*n* = 54		
Median RT	47.02	50.09	1.287	0.202
Comission errors	45.43	53.00	3.381	*0.001*
Omissions	46.53	50.20	1.691	0.095

Statistically significant figures are presented in italics. RT: Reaction time; SD: standard deviation.

### Linear regression models of cognitive tests

Belonging to the ADHD group in age-adjusted Model 1 predicted commission errors in Divided Attention (Adj. R. sqr. 0.068, B= −0.294, 95% CI −10.115; −2.496, *p=*.001), Flexibility (Adj. R sqr. 0.140, B= −0.405, 95% CI −17.121; −4.396, *p=*.001) and Distractibility (Adj. R sqr. 0.106, B= −0.354, 95% CI −15.296; −2.811, *p=*.005), as well as omission errors in Inhibitory Control (Adj. R sqr. 0.180, B= −0.404, 95% CI −13.959; −2.293, *p=*.008). Belonging to the ADHD group also predicted longer reaction times (Adj. R sqr. 0.090, B= −0.304, 95% CI −8.696; −0.929, *p=*.017) and larger standard deviation of reaction time (SD) (Adj. R sqr. 0.181 B= −0.434, 95% CI −13.386; −4.003, *p<*.001) in Flexibility.

In the fully adjusted Model 2 (adjusted for age, sex and IQ) of linear regression, belonging to the ADHD group predicted commission errors in Divided Attention (Adj. R sqr. 0.092, B= −0.275, 95% CI −9.732; −2.092, *p=*.003), Flexibility (Adj. R. sqr. 0.094, B= −0.262, 95% CI −10.496; −1.870, *p=*.005), Distractibility (Adj. R sqr. 0.184, B= −0.333, 95% CI −14.394; −2.432, *p=*.007) and Sustained Attention (Adj. R sqr. 0.211, B= −0.402, 95% CI −13.082; −3.492, *p=*.001), as well as omission errors in Inhibitory Control (Adj. R sqr. 0.269, B= −0.399, 95% CI −13.557; −2.522, *p=*.005) and a larger SD in Flexibility (Adj. R sqr. 0.137, B= −0.270, 95% CI −7.808; −1,596, *p=.*003).

### Group differences in facial emotion recognition

The total number of correct answers in facial emotion recognition was generally high. Mean correct answers in the Facial emotion recognition task for the ADHD group was 98.52 (82.1%) and for the control group 98.50 (82.1%) out of 120 items. In the Sentence matching task, the ADHD group scored 34.34 (81.8%) and controls 34.51 (82.2%) out of 42 items, respectively. The ease of recognizing emotional expressions varied according to the emotion category. In the Facial emotion recognition task, the sequence for correct answers in the ADHD group was as follows: Happy > Neutral > Disgust > Sad > Fear > Anger; and in the control group: Happy > Neutral > Disgust > Sad > Anger > Fear. In the Sentence matching task, the sequence of correct answers in the ADHD group was: Disgust > Sad > Fear > Happy > Anger > Surprise; and in the control group: Sad > Disgust > Fear > Happy > Anger > Surprise (Table 3).

**Table 3.** Facial affect recognition group difference (Student T-test).

	ADHD mean (SD)	Control mean (SD)	Test statistics	*p*-value
Facial emotion recognition task				
	*n* = 59	*n* = 69		
Emotion recognition total				
Corrects	98.51 (12.03)	98.13 (12.11)	−0.38	0.860
Reaction time	1784.71 (465.47)	1905.53 (490.38)	0.06	0.137
Fear				
Corrects	15.95 (2.86)	15.07 (3.03)	−0.88	0.097
Reaction time	1797.67 (509.92)	1942.99 (497.63)	0.08	0.079
Disgust				
Corrects	16.78 (2.75)	16.45 (2.56)	−0.33	0.485
Reaction time	1708.31 (459.55)	1844.43 (462.06)	136.12	0.098
Happy				
Corrects	17.76 (2.06)	18.07 (2.18)	0.31	0.413
Reaction time	1666.18 (467.10)	1722.66 (427.99)	0.04	0.393
Sad				
Corrects	16.51 (2.51)	16.32 (3.05)	−0.19	0.705
Reaction time	1840.32 (548.84)	1929.71 (575.13)	89.39	0.372
Anger				
Corrects	14.47 (2.78)	15.20 (2.05)	0.73	0.091
Reaction time	1831.81 (514.31)	1964.74 (538.68)	132.93	0.158
Neutral				
Corrects	17.03 (2.30)	16.81 (2.91)	−0.22	0.637
Reaction time	1864.00 (513.85)	2043.93 (659.01)	0.08	0.111
Sentence matching task				
	*n* = 60	*n* = 66		
Sentence matching total				
Corrects	34.32 (3.67)	34.24 (3.34)	−0.07	0.906
Reaction time	6346.26 (1315.10)	6625.61 (1351.79)	279.34	0.243
Fear				
Corrects	6.02 (1.03)	5.89 (1.01)	−0.12	0.502
Reaction time	6344.24 (1630.75)	6695.86 (1530.19)	351.62	0.214
Disgust				
Corrects	6.30 (1.09)	6.27 (1.07)	−0.03	0.888
Reaction time	6188.64 (1656.38)	6409.66 (1647.50)	221.02	0.455
Happy				
Corrects	5.85 (0.88)	5.77 (1.05)	−0.08	0.657
Reaction time	6254.81 (1598.41)	6363.99 (1540.24)	109.18	0.697
Sad				
Corrects	6.28 (1.03)	6.29 (0.94)	−0.11	0.956
Reaction time	5989.71 (1246.95)	6123.62 (1713.28)	0.01	0.785
Anger				
Corrects	5.42 (1.43)	5.52 (1.19)	0.10	0.674
Reaction time	6264.12 (1544.19)	6881.08 (1506.61)	616.96	0.025
Surprise				
Corrects	4.45 (1.36)	4.50 (1.46)	0.05	0.843
Reaction time	7030.84 (1506.29)	7259.95 (1556.72)	229.11	0.404

Statistically significant figures are presented in **bold**. RT: reaction time, SD; standard deviation.

Overall reaction times and the recognition capacity of all the presented emotions in the Facial emotion recognition task were alike in the two groups (Table 3). When comparing the recognition of negative emotions (Fear, Disgust, Sadness, Anger) between the ADHD group and the control group, we observed no group differences (t=-0.446, *p=*.657). Similarly, no group differences were observed regarding the recognition of positive/neutral (Happy, Neutral) emotions (*t* = 0.116, *p=*.908).

The two groups exhibited equal reaction times and recognition capacity when examining summary results of all the presented emotions in the Sentence matching task as well. The ADHD group displayed, however, faster reaction times in the recognition of Anger *(p=*.025) (Table 3). No group differences were observed in the recognition of negative (Fear, Disgust, Sadness, Anger) (t= −0.099, *p=*.922) or positive/neutral (Happy, Surprised) (t= −0.087, *p=*.091) emotions.

### Linear regression models of facial emotion recognition tests

Linear regression analyses adjusted for age showed that belonging to the ADHD group was associated with less correct answers in the recognition of Anger (Adj. R sqr. 0.035, B= −0.918, 95% CI −1.778; −0.059, *p=*.037) in the Facial emotion recognition task. However, group status had no contribution to the facial emotion recognition variables when controlled for age, IQ and sex in linear regression Model 2 (Supplementary Table 2). Borderline significant result was still observed for the recognition of Anger (Adj. R sqr. 0.029, B= −0.879, 95% CI −1.7630;0.004, *p*=.051). Age remained a significant predictor for facial emotion recognition in both the Facial emotion recognition task (Adj. R sqr. 0.071, B= −11.832, 95% CI −20.386; −3.278, *p=*.007) and the Sentence matching task (Adj. R sqr. 0.115, *B* = 0.626, 95% CI 0.298;0.953, *p<*.001) along with reaction time in the Facial emotion recognition task (Adj. R sqr. 0.243, B= −0.065, 95% CI −0.086;-0.044, *p<*.001).

Linear regression model 3 showed significant contribution of interaction terms (cognitive test x group) for Divided Attention commission errors x Fearful faces (Adj. R sqr. 0.131, *B* = 0.885, 95% CI 0.001;0.197, *p=*.048), Sustained Attention commission errors x Disgust sentences (Adj. R sqr. 0.025, B= −1.202, 95% CI 0.109; −0.001, *p=*.046), Inhibitory Control omission errors x Disgust sentences (Adj. R sqr. 0.028, *B* = 1.133, 95% CI 0.003;0.107, *p=*.037) and Inhibitory Control omission errors x Surprise sentences (Adj. R sqr. 0.057, *B* = 1.087, 95% CI 0.003;0.139, *p=*.042).

Further analyses, where the sample was divided by group status (i.e. ADHD/control group) displayed that, in the ADHD group, committing fewer errors in the Divided Attention test significantly predicted correct answers in overall facial emotion recognition (Adj. R sqr. 0.198, *B* = 0.347, 95% CI 0.079;0.606, *p=*.012), in recognition of fearful (Adj. R sqr. 0.144, *B* = 0.348, 95% CI 0.017;0.148, *p=*.014), and in recognition of neutral faces (Adj. R sqr. 0.175, *B* = 0.322, 95% CI 0.011;0.125, *p=*.021). No prediction value for cognitive test performance was observed in the control group facial emotion recognition or Sentence matching task performance.

### Effect sizes

In both the facial emotion recognition task and the sentence task the group differences in correct responses were negligible (*d* = 0.031; *d* = 0.023), while small effect sizes were observed for group differences in reaction times (*d* = 0.288; *d* = 0.209), respectively.

## Discussion

As expected, children with ADHD showed more behavioural issues and performed worse in cognitive tests compared to typically developing children. However, our hypothesis suggesting that typically developing children outperform children with ADHD in facial emotion recognition tests was not confirmed. The ADHD group answered significantly faster (and equally correct) to facial emotional expressions of anger in the Sentence matching task. However, when controlling for differences in age, sex and IQ, we found no significant group differences in either of the facial emotion recognition tasks.

While the sequences for observing different emotions in Emotion recognition and Sentence matching task did not vary markedly between the ADHD and Control groups, we observed a difference between the sequences of recognised emotions reported between the two tasks. This possibly reflects the more subjective nature of the Sentence matching task, compared to the simple emotion recognition task, as it is more open to personal interpretations and associations of the depicted situations. Task design therefore appears imperative for the outcome, indicating that facial emotion recognition tasks should be selected carefully when designing future studies.

Similarly to one other study [19], our study was restricted to school aged children. In our sample, the ADHD group was on average eight months older than the control group. This could have influenced our overall results, since age emerged as a significant predictor for facial emotion recognition in both tasks. A finding that is supported by Rinke and colleagues (2017), whose results indicate that the processes underlying facial emotion recognition are primarily age dependent.

Of the previous studies that have investigated the ability to recognize facial emotions in individuals with ADHD our findings are in concordance with the third line of research [17–23, 2021). We did not observe a difference in emotion recognition capacity between children with ADHD and children of the typically developing control group. Yet we observed also faint similarities with the first line of research [6–8, 10] suggesting that deficits in recognising facial expressions represent a secondary negative effect resulting from primary cognitive impairments. In our data cognitive functions (i.e. better performance in a test assessing divided attention) appeared to predict facial emotion recognition – but solely in the ADHD group. This might indicate a link between cognitive functions and facial emotion recognition within ADHD populations. Future studies should further clarify this finding by stratifying ADHD groups according to performance in a test assessing divided attention, and possibly also other cognitive parameters.

The association of emotion recognition tasks with cognitive functions in both groups furthermore underline the importance of cognition in the recognition of facial emotions. Our findings are in line with previous studies [9,18,23,25] as we found significant associations between emotion recognition tasks and tests assessing divided attention and inhibitory control.

As expected, questionnaires assessing ADHD symptomatology, behaviour, executive functions and children’s individual strengths and difficulties showed significant group differences [45]. We did not find significant correlations between oppositionality and identification of facial emotions in either of the emotion recognition tests. The previous suggestions that processes leading to oppositionality also explain the possible emotion recognition deficit in ADHD [6,20,22] is not supported by our study. Similarly, Anxious/Depressed and Social problem CBCL scores were uncorrelated with the identification of facial emotions.

In cognitive tests, the greater amount of omission and commission errors in the ADHD group may indicate impairments in executive functioning. These findings are in line with previous research in ADHD populations, which reported impairments in cognitive flexibility and inhibitory control [32,34,46]. In particular, our observation that group differences in a number of attention/executive functions (i.e. divided attention, distractibility, response inhibition, cognitive flexibility and sustained attention) remain significant after controlling for age and IQ, highlight the importance of considering differences in cognitive performance between children with ADHD and typically developing children. This view is also supported by findings of a positive correlation between cognitive flexibility and facial emotion recognition in children with DMDD and comorbid ADHD [24].

Several factors may explain heterogeneous findings in emotion recognition of ADHD populations. Firstly, studies have been using varying facial emotion recognition materials and testing procedures, which precludes direct comparison of results. For the present study, for example, we utilised an in-house developed facial recognition paradigm. Secondly, the sex distribution might affect outcomes. Studies with samples consisting of only boys appear to have consistently detected group differences between ADHD and control groups in facial emotion recognition tests [10,13,47] while the findings in mixed-sex samples vary. Thirdly, IQ is known to affect facial emotion recognition [48]. In the present study, the mean IQ in the ADHD group was six points lower than in the control group. Consequently, we adjusted our regression model for age, sex and IQ in order to control the influence of these factors on facial emotion recognition.

The following limitations should be considered while interpreting our results. First, we did not control for comorbidities or divide the ADHD group based on symptoms presentations. Our goal was to utilise a naturalistic sample that best represents the clinical environment, and we did not intend to differentiate ADHD presentations according to facial emotion recognition skills. However, it would be interesting to elucidate the role of comorbid symptoms in a future trial. Second, upon entering the study, diagnoses other than ADHD were established clinically but not confirmed with standardized clinical interviews. Nevertheless, our questionnaire results strongly supported the prior diagnoses set. Third, when interpreting our findings, it is important to take into consideration that we had an ADHD group in which patients with significant social problems were excluded. This limits the generalizability to average clinical samples of ADHD patients. Finally, in the Sentence matching task, the presentation time of the facial photo was not fixed. Facial emotion recognition is a fast process, usually <200 ms [27,49]. Self-paced responding might have given a child with ADHD the needed extra time to orientate properly. Notably, self-pacing appeared not to promote impulsive behaviour, as reaction times between the two groups did not differ.

We found no significant differences in the facial emotion recognition capacity between children with ADHD and a group of typically developing children. Cognitive performance in a test assessing divided attention appears to predict the capacity for facial emotion recognition in ADHD.

## Supplementary Material

20240723_Supplementary_File.docx
