# Supplementary material for: Facial emotion recognition in children with attention deficit hyperactivity disorder
Source: Nord J Psychiatry. 2024 Sep 18;78(7):634–43. doi: 10.1080/08039488.2024.2403589 (PMC11458127; doi:10.1080/08039488.2024.2403589)
Supplement: 20240723_Supplementary_File.docx [file IPSC_A_2403589_SM0923.docx]

**Supplementary Materials**

*Correlation of emotion recognition with cognitive test results*

In the ADHD group, the overall emotion recognition number of correct responses correlated with Divided attention commission errors (r=0.407, *p=*.003; z=1.841, *p*=0.033) and Sustained attention commission errors (r=0.404, *p=*.030; z=1.64, *p*=0.05), and the Sentence matching task overall number of correct responses correlated with Divided attention median reaction time (r=0.323, *p=*.015; z=-1.018, *p*=0.154) and Sustained attention omission errors (r=-0.374, *p=*.050; z=-1.702, *p*=0.044). In the Control group, the overall emotion recognition number of correct responses correlated with Inhibitory Control reaction time standard deviation (r=0.523, p<.001; z=-2.301, *p*=0.011) (**Supplementary Table 1**).

**Supplementary Table 1.** Correlations of behavioural and cognitive test results with emotion recognition

| ADHD | | Oppositional Defiant | Divided Attention reaction time | Divided Attention comission errors | Inhibitory Control reaction time | Inhibitory Control reaction time SD | Inhibitory Control comission errors | Inhibitory Control omission errors | Flexibility reaction time | Flexibility reaction time SD | Flexibility comission errors | Sustained Attention reaction time | | Sustained Attention comission errors | Sustained Attention omission errors |
| --- | --- | --- | --- | --- | --- | --- | --- | --- | --- | --- | --- | --- | --- | --- | --- |
| Emotion Recognition Corrects | **r** | -0.137 | 0.189 | 0.407 | 0.030 | 0.151 | 0.061 | 0.094 | -0.196 | 0.036 | 0.211 | -0.112 | 0.404 | | 0.070 |
|  | ***p*** | 0.349 | 0.167 | **0.003** | 0.824 | 0.264 | 0.654 | 0.496 | 0.160 | 0.803 | 0.130 | 0.563 | **0.030** | | 0.719 |
|  | **N** | 49 | 55 | 52 | 56 | 57 | 56 | 55 | 53 | 51 | 53 | 29 | 29 | | 29 |
| Sentence Task Corrects | **r** | -0.139 | 0.323 | 0.080 | 0.194 | 0.138 | -0.052 | 0.113 | -0.101 | 0.160 | 0.052 | 0.141 | -0.027 | | -0.374 |
|  | ***p*** | 0.335 | **0.015** | 0.571 | 0.149 | 0.300 | 0.701 | 0.408 | 0.469 | 0.258 | 0.709 | 0.475 | 0.892 | | **0.050** |
|  | **N** | 50 | 56 | 53 | 57 | 58 | 57 | 56 | 54 | 52 | 54 | 28 | 28 | | 28 |
| Control |  |  |  |  |  |  |  |  |  |  |  |  |  | |  |
| Emotion Recognition Corrects | **r** | -0.060 | 0.126 | 0.076 | -0.006 | 0.523 | 0.175 | -0.071 | -0.074 | 0.103 | 0.200 | -0.038 | 0.029 | | 0.078 |
|  | ***p*** | 0.645 | 0.329 | 0.559 | 0.962 | **0.000** | 0.173 | 0.584 | 0.570 | 0.436 | 0.120 | 0.792 | 0.839 | | 0.588 |
|  | **N** | 61 | 62 | 62 | 62 | 65 | 62 | 62 | 61 | 59 | 62 | 51 | 51 | | 51 |
| Sentence Task Corrects | **r** | -0.034 | -0.139 | 0.037 | -0.094 | 0.146 | 0.173 | 0.015 | 0.037 | 0.170 | 0.162 | -0.068 | -0.043 | | 0.035 |
|  | ***p*** | 0.797 | 0.293 | 0.777 | 0.473 | 0.248 | 0.183 | 0.906 | 0.781 | 0.210 | 0.217 | 0.654 | 0.775 | | 0.817 |
|  | **N** | 59 | 59 | 61 | 61 | 64 | 61 | 61 | 59 | 56 | 60 | 46 | 46 | | 46 |

*Statisticially significant results (p≤..05) in* ***bold****,* *r; Pearson Correlation coefficient, SD; Standard deviation. Note that the sustained attention task only includes KITAP results (children aged <11 years).*

**Supplementary Table 2.** Linear regression for the capacity of the group status to predict facial emotion recognition (analyses controlled for age, IQ and gender).

| **Variable** | **Adjusted R square** | **Estimate** | **95% CI** | **Linear regression P** |
| --- | --- | --- | --- | --- |
| **Facial recognition Correct** | 0.206 | -0.402 | (-4.357;3.553) | 0.841 |
| **Facial recognition Median RT** | 0.283 | -0.025 | (-0.097;0.046) | 0.482 |
| **Sentence matching Correct** | 0.115 | 0.051 | (-1.172;1.273) | 0.934 |
| **Sentence matching Median RT** | -0.002 | -363.703 | (-844.474;117.068) | 0.137 |
| **Disgust Correct** | 0.176 | 0.066 | (-0.823;0.956) | 0.883 |
| **Disgust Median RT** | 0.229 | -96.735 | (-225.894;32.424) | 0.141 |
| **Fear Correct** | 0.105 | 0.681 | (-0.358;1.72) | 0.197 |
| **Fear Median RT** | 0.189 | -0.04 | (-0.121;0.041) | 0.330 |
| **Happy Correct** | 0.166 | -0.46000 | (-1.176;0.255) | 0.205 |
| **Happy Median RT** | 0.226 | 0.0230 | (-0.052;0.098) | 0.549 |
| **Sad Correct** | 0.14 | 0.311 | (-0.0649;1.271) | 0.522 |
| **Sad Median RT** | 0.23 | 0.004 | (-0.0820;0.089) | 0.935 |
| **Anger Correct** | 0.029 | -0.879 | (-1.7630;0.004) | 0.051 |
| **Anger Median RT** | 0.206 | -16.365 | (-158.983;126.253) | 0.821 |
| **Neutral Correct** | 0.18 | 0.067 | (-0.815;0.948) | 0.881 |
| **Neutral Median RT** | 0.276 | -0.036 | (-0.121;0.05) | 0.409 |
| **Sentence Disgust Correct** | 0.018 | -0.053 | (-0.451;0.346) | 0.794 |
| **Sentence Disgust Median RT** | -0.014 | -180.086 | (-793.953;433.78) | 0.562 |
| **Sentence Fear Correct** | 0.035 | 0.098 | (-0.274;0.471) | 0.602 |
| **Sentence Fear Median RT** | 0.006 | -527.412 | (-1092.329;37.505) | 0.067 |
| **Sentence Happy Correct** | 0.063 | 0.168 | (-0.181;0.518) | 0.342 |
| **Sentence Happy Median RT** | -0.011 | -88.892 | (-661.669;483.885) | 0.759 |
| **Sentence Sad Correct** | 0.068 | -0.215 | (-4.117;3.687) | 0.913 |
| **Sentence Sad Median RT** | 0.007 | -0.023 | (-0.11;0.063) | 0.593 |
| **Sentence Anger Correct** | -0.002 | -0.159 | (-0.646;0.329) | 0.52 |
| **Sentence Anger Median RT** | 0.03 | -206.618 | (-783.277;370.04) | 0.479 |
| **Sentence Surprise Correct** | 0.049 | 0.04 | (-0.471;0.551) | 0.877 |
| **Sentence Surprise Median RT** | 0.002 | -462.13 | (-1052.407;128.147) | 0.124 |

RT; Reaction time, CI; Confidence interval

**Distribution of ADHD presentations**:

Combined presentation (n=43, 70,5 %), Inattentive presentation (n=15, 24.6 %), Hyperactive-impulsive presentation (n=3, 4.9 %).
